# Supplementary material for: Patatin-Related Phospholipase pPLAIIIγ Involved in Osmotic and Salt Tolerance in Arabidopsis
Source: Plants (Basel). 2020 May 20;9(5):650. doi: 10.3390/plants9050650 (PMC7284883; doi:10.3390/plants9050650)
Supplement: Supplementary file 1 [file plants-09-00650-s001.zip › Figure S1, 2.docx]

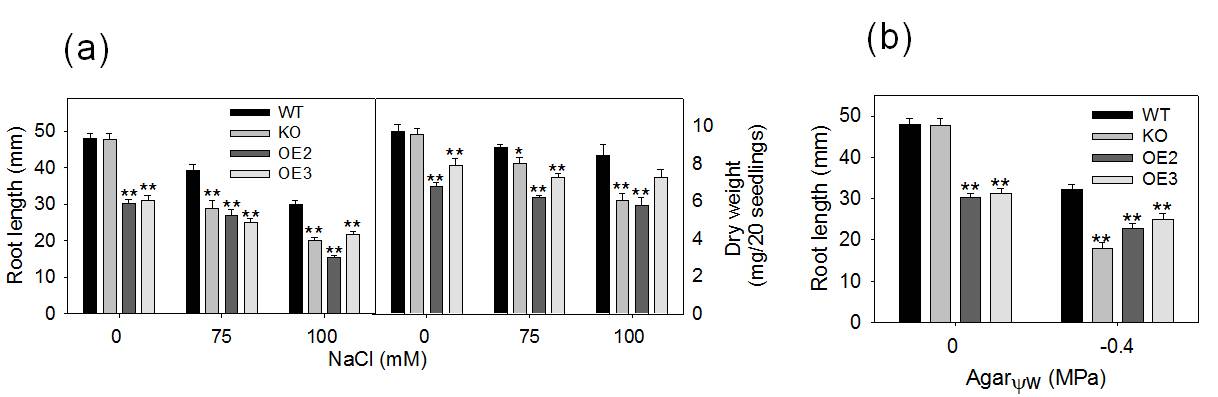


**Figure 1.** Root length and dry weight of WT, KO, and OE‑lines under salt and PEG stresses. (**a**) Measurement of root length and dry weight. Seedlings were grown for 7 days on plates in a half‑strength MS medium with 0, 75, 100 mM NaCl after transferring. Values are mean ± SE (*n* = 45—60) for root length, error bars indicate the SE (n = 3) for dry weight. (**b**) Measurement of root length under PEG stress. Seedlings were grown, harvested and qualified as (**a**). Asterisk presents a significant difference on Student’s test (*p* = 0.05).


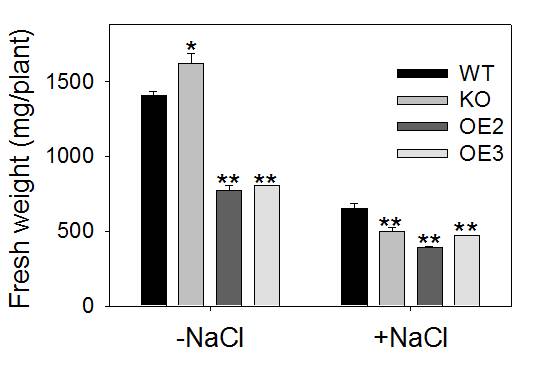


**Figure 2.** Plant tolerance to salt in soil. Three‑week‑old WT, *pPLAIIIγ‑*KO, and OE plants grown in the same tray were watered with 0 or 100 mM NaCl for 20 days. The above‑ground weight were measured. Data represent the means ± SE (*n* = 10—12). Asterisk represents a significant difference at 0.05 level by Student’s test.
