# Supplementary material for: Patatin-Related Phospholipase pPLAIIIγ Involved in Osmotic and Salt Tolerance in Arabidopsis
Source: Plants (Basel). 2020 May 20;9(5):650. doi: 10.3390/plants9050650 (PMC7284883; doi:10.3390/plants9050650)
Supplement: Supplementary file 1 [file plants-09-00650-s001.zip › Legends for the supplemental Excel files.docx]

Legends for the Supplemental Excel Files

Excel 1 Gene Expression Data

Sheet 1. Relative gene expression of pPLAIIIγ in WT, KO, OE, and COM lines by qPCR (Figure 1c).

Sheet 2. Transcript level of pPLAIIIγ measured by qPCR in leaves and roots of WT with and without 100 mM NaCl at different time points (1—24 h) (Figure 7).

Sheet 3. Transcript levels of SOS2 quantified by qPCR in roots of WT and KO seedlings with 0 or 100 mM NaCl treatments at different time points (1—24 h) (Figure 7).

Sheet 4. Transcript levels of SOS3 quantified by qPCR in roots of WT and KO seedlings with 0 or 100 mM NaCl treatments at different time points (1—24 h) (Figure 7).

Sheet 5. Transcript levels of AtCBL10 quantified by qPCR in roots of WT and KO seedlings with 0 or 100 mM NaCl treatments at different time points (1—24 h) (Figure 7).

Excel 2 Osmotic and Salt Stress Data

Figure 1e. Measurement of primary root length of WT, KO, and COM lines under NaCl stresses.

Figure 1f. Measurement of primary root dry weight of WT, KO, and COM lines under NaCl stresses.

Figure 2b. Quantification of root length in response to different levels of sorbitol.

Figure 2c. Quantification of dry weight in response to different levels of sorbitol.

Figure 2d. Quantification of root length of WT and KO lines in response to different levels of PEG.

Figure 2e. Quantification of root length of WT, COM and KO lines in response to different levels of PEG.

Figure 3f. The relative growth of root length of WT, KO, and OE lines in response to NaCl and PEG.

Figure 3g. The relative growth of dry weight of WT, KO, and OE lines in response to NaCl.

Figure 4b. Effect of NaCl on seed germination phenotype of wild type (WT), pPLAIIIγ knockout (KO), and OE‑lines under salt stress.

Figure 5b. Measurement of the relative growth of WT, KO, and OE lines in soil under salt and drought stress.

Figure 5c. Measurement of wilted and survived plants when water was withdrawn (Wilted), and survived plants that wilted plants were watered again when water was resumed (Survived).

Excel 3 FFA and Lysolipids Data

FFA: Effects of pPLAIIIγ‑KO and OE on free fatty acids (FFA) levels in Arabidopsis.

Lysolipids: Effects of pPLAIIIγ‑KO and OE on lysophospholipid (Lysolipid) levels in Arabidopsis.
