# Supplementary material for: Patatin-Related Phospholipase pPLAIIIγ Involved in Osmotic and Salt Tolerance in Arabidopsis
Source: Plants (Basel). 2020 May 20;9(5):650. doi: 10.3390/plants9050650 (PMC7284883; doi:10.3390/plants9050650)
Supplement: Supplementary file 1 [file plants-09-00650-s001.zip › Table S1.docx]

**Table S1.** Primer information in this study.

| **Gene Name** | **Primer Name** | **Sequence (5′ to 3′)** |
| --- | --- | --- |
| pPLAIIIγ KO | LP | TATCATCGCAGCGAATACTCC |
|  | RP | TTGGAATCCATTTGGGAAAAG |
| LBb1.3 | BP | GCGTGGACCGCTTGCTGCAACT |
| pPLAIIIγ OE | JLP023 | CCTTAATTAAATGAATCGGCGCTACGAAAAGC |
|  | JLP024 | TTGGCGCGCCTCTATCTTTAGATATGAGAGTG |
| pPLAIIIγ Com | JLP027 | TTGGCGCGCCTGACTTGAAACCATCCAATCTG |
|  | JLP028 | TTGGCGCGCCGTGCAAGAAACAGGTCTCACTC |
| UBQ10 | JLP029 | CACACTCCACTTGGTCTTGCGT |
|  | JLP030 | TGGTCTTTCCGGTGAGAGTCTTCA |
| pPLAIIIγ | JLP053 | GCAAAAGGATAGGAGAAATGAG |
|  | JLP054 | TTTAGCGTTACGGTTGGAGA |
| AtSOS2 | JLP041 | AAGCTATGTTCGAAACTGGAAAAC |
|  | JLP042 | TGGATTTAAGTTGGGATCAAAACG |
| AtSOS3 | JLP043 | AGAAGGGTGTGTTTGTATGGG |
|  | JLP044 | GAAGCTCGGGATCCTCATATC |
| AtCBL10 | JLP045 | ACGACAGCATTCCCAAGTT |
|  | JLP046 | CCGGTGTTGCATGGACAG |
